# Reasons behind individuals’ self-ratings of health: an analysis of responses to an open-ended survey question

**DOI:** 10.1186/s12889-026-27905-0

**Published:** 2026-05-23

**Authors:** Michael Mutz, Johannes Müller

**Affiliations:** 1 Institute of Sport Science, Justus-Liebig-Universität Giessen, Kugelberg 62, Giessen, 35394 Germany; 2 Institute of Sport and Movement Science, Universität Vienna, Auf der Schmelz 6, Vienna, 1150 Austria

**Keywords:** Biopsychosocial model, Mental health, Mixed methods, Subjective health

## Abstract

**Background:**

Self-rated health (SRH) is one of the most widely used indicators in population health research, yet the meanings respondents attach to this global item remain insufficiently understood. This study examines how individuals justify their SRH assessments and whether the domains invoked vary systematically by health status and sociodemographic position. The aim was to clarify what respondents mean when they rate their health and to identify patterns in the salience of bodily, psychological, social, and lifestyle-related aspects of health.

**Methods:**

We used a mixed-methods design based on representative survey data that combined a standard SRH item with an open-ended follow-up question asking respondents to explain their rating in their own words. Open responses were coded into thematic health domains and analyzed quantitatively across subgroups defined by age, gender, income, and health status.

**Results:**

SRH emerged as a domain-weighted judgment. Acute bodily complaints and chronic conditions were the most common justifications overall, particularly among respondents in poorer health. In contrast, “very good” health ratings were more often linked to emotional well-being, social relations, and broader functioning. Older respondents referred more frequently to chronic disease burden and functional capacity, whereas younger respondents more often emphasized acute symptoms and social relations. Women gave greater weight to affective states and caregiving responsibilities. Higher-income respondents referred less to chronic disease and more to emotional well-being than lower-income respondents.

**Conclusions:**

SRH should be interpreted neither as a purely biomedical indicator nor as a uniformly integrated biopsychosocial assessment. Its meaning is context-dependent and shaped by respondents’ health status, social roles, and socioeconomic position. This has implications for the interpretation and comparability of SRH across population groups.

**Supplementary Information:**

The online version contains supplementary material available at 10.1186/s12889-026-27905-0.

## Background

The subjective health question (“*How in general would you rate your health*?”) is a key single-item indicator in numerous large-scale social and health surveys. It is included, for instance, in the International Social Survey Programme’s health and healthcare module (https://issp.org/), the cross-national Health Behavior in school-aged Children studies (https://www.hbsc.org/), or the U.S. National Health Interview Survey (https://www.cdc.gov/nchs/nhis/index.html). Health researchers conceive this indicator as a cognitive-affective summary judgment that people form by integrating multiple cues about how their body feels and functions, how they are coping psychologically, and how well they are able to live their lives in their social context [1–3].

Reliability analyses indicate that self-rated health (SRH) has a good overall test-retest reliability [4]. A large body of literature shows that SRH predicts morbidity and mortality even after accounting for many objective clinical indicators [5–7]. In addition, improving and deteriorating SRH over time is also predictive of mortality among aging individuals [8]. Studies show that SRH is closely associated with a large variety of medical conditions and diseases [9–13] and predicts future hospitalization records [14]. It was even suggested that the single SRH item captures almost the same amount of information than complex measures and symptoms checklists [15]. Scholars have argued that SRH may not only incorporate a wide range of information, but also reflect how this information is evaluated in its dynamic nature and in relation to a person’s ability to cope [16]. This makes SRH a useful, time-efficient indicator for capturing broad health information with minimal data collection burden.

From a biopsychosocial health perspective, a person’s overall state of health and functioning emerges from the dynamic interaction of (1) biological factors (e.g., physiology, disease processes, neuroendocrine and immune function), (2) psychological factors (e.g., emotions, cognition, coping, mental health), and (3) social factors (e.g., relationships, social support, socioeconomic conditions, work and community contexts). In this view, health, as a multidimensional construct, includes well-being, functioning, and adaptability across these three interdependent domains. This framing is most associated with the biopsychosocial model [17] and aligns with broader conceptions of health and functioning put forward by the World Health Organization [18]. Although scholars conceive SRH as a judgement informed by biological, psychological and social factors [1], few studies have yet tried to analyze inasmuch each factor or their specific combination influences the overall rating.

In her narrative review, Garbarski [1] summarized the large number of quantitative studies that have examined the associations between physical and mental health conditions and SRH. Most of these studies found that physical parameters are among the most influential determinants of SRH, including acute and chronic diseases, pain, weight status, medications and functional impairments [5, 12, 13, 19–22]. Psychological factors, such as stress, financial worries or critical life events, can also predict SRH [19, 23, 24]. Research further suggests that lifestyle factors are important predictors of SRH, with physical activity being positively associated with SRH [25, 26] and smoking negatively associated with it [27]. All of these studies relate SRH scores to a predefined set of predictors. While this hypothesis-driven approach is well suited to testing specific associations, it may overlook aspects that respondents themselves consider central to their ratings. Garbarski [1] and Jylhä [5] therefore called for research that examines the cognitive processes underlying subjective health ratings in detail, for instance, how people – as individuals or as representatives of social groups – reason about their health and which considerations they prioritize when forming a global assessment.

A number of existing studies have examined the reasons underlying SRH and found meaningful differences, for instance, according to age, gender, ethnicity, or education [2, 20, 28–34]. These studies have employed a range of methods to capture such reasons. Some used open-ended probes immediately following the SRH rating, asking respondents to provide a written justification for their assessment [28–30]. Others drew on semi-structured qualitative interviews to reconstruct respondents’ broader understandings of health [2, 31, 32]. A third strand applied a ‘think-aloud’ approach, in which respondents verbalize their thoughts while completing a questionnaire [33, 34].

Findings point to a declining importance of acute diseases and health behaviors for SRH over the life-course, while the influence of functioning, chronic conditions and medical treatments increases [20, 28, 31]. In addition, psychological references to well-being are more prevalent among younger age groups [31]; including school-related stress and insecurity among youths [33]. Besides age, also gender and socio-economic status (SES) influence the reasoning behind SRH. Men justify their health ratings more frequently with physical functioning or (negative) health behaviors than women [28]. For young mothers, family functioning, emotional wellbeing, energy and tiredness were central themes related to health judgements [34]. Higher educated individuals were more likely to mention physical functioning and vitality as reasons for SRH [28], whereas lower educated individuals were more likely to mention health behaviors [31]. Reference groups that respondents have in mind when assessing their health, such as friends, colleagues or “sick people”, also figure in and influence the rating [30]. Overall, scholars have concluded that SRH has different meanings in different population groups [1, 28, 31]. However, a study comparing Germany, Great Britain, the U.S., Mexico and Spain indicates that respondents from these countries had largely similar reasons in mind, when judging their health [29].

While qualitative interview studies are usually well suited for exploring deeper layers of meanings, their shortcomings are the small, non-representative samples that often do not allow to compare subgroups. In addition, some studies using probes were also based on non-representative convenience samples that do not allow for generalizing findings to populations [28, 30]. The present study responds to this deficit by eliciting respondents’ rationales through an open-ended prompt administered as part of a population-wide survey. Using representative survey data, our design combines the interpretive breadth of open-ended responses with the advantage of a large-scale sample, thereby strengthening generalizability of findings. Moreover, our research addresses a substantive gap in the literature by examining whether individuals draw on physical, mental, and social factors when assessing their health, and whether they consider these domains simultaneously. While prior research has shown that self-assessments of health are shaped by multiple dimensions, the question inasmuch respondents combine different domains remains insufficiently understood.

Methodologically, we build on existing studies that have used probing techniques to elicit respondents’ subjective rationales for previously provided answers [28–30]. In the context of SRH, the written reasons and justifications allow us to identify the information that most strongly informs the response, including considerations that may not be captured by conventional medical indicator sets. We analyze these accounts in several respects. First, we assess the extent to which respondents invoke biological, psychological, and social considerations when justifying their overall health ratings. Second, we examine whether the content and emphasis of these justifications vary by age, gender, socio-economic status (SES), and health rating. Third, exploiting the qualitative data, we searched for additional patterns in the open-ended responses that help to understand the reasoning behind SRH.

## Methods

This study draws on an open-ended survey question designed to capture respondents’ reasons and justifications for a preceding SRH assessment. Respondents first answered the standard SRH question, “*How is your health in general*”, with response options ranging from “very poor” and “poor” to “fair,” “good,” and “very good.” Immediately afterwards, respondents were asked to briefly explain why they selected a particular rating. Precisely, they were asked: “*You have just rated your health as […]. What are the most important reasons for this self-assessment?*” (see also Supplement). Similar probes have been used by previous studies [28–30]. Their main advantage is the capacity of making visible the considerations, interpretations, and evaluative criteria that underlie survey responses, while allowing respondents to articulate these in their own words rather than within predefined response categories.

The item was embedded in a large-scale representative survey of the German population aged 18 years and older (*N* = 1,006). Data collection was conducted in cooperation with Forsa, a German institute specializing in public opinion research. Forsa maintains a nationally representative panel, based on probability sampling standards; self-selection into the panel is not possible. The raw sample closely reflects the German adult population with regard to age composition (18–29 years: 15%; 30–44: 24%, 45–59: 25%; ≥60: 35%), gender (female: 51%; male: 49%), and region of residence (Eastern Germany: 15%; Western Germany: 85%). The survey itself was designed as a multi-topic study, allowing research institutions, media outlets, and companies to contribute questions of interest. In the survey wave analyzed here, the health-related question was fielded next to questions on religiosity and church attendance. While a multi-topic survey is, by design, thematically less focused than a topic-specific instrument, it offers the methodological advantage of mitigating response selectivity: a broader range of topics ensures that participation is not driven by a particular thematic interest, thereby reducing the risk that only respondents with a strong affinity for a specific subject feel addressed and choose to take part.

Participants were able to complete the questionnaire online using a computer, tablet, or smartphone. This mode of data collection is particularly advantageous for open-ended questions, as it gives respondents time to formulate their answers in their own words and may reduce potential interviewer effects. A total of 852 from 1,006 respondents provided a valid answer to the open-ended question. These responses averaged 7.2 words and ranged from very brief one-word answers (e.g., “diabetes” or “depression”) to longer, more detailed accounts describing personal health trajectories, sources of psychological suffering, or a person’s broader life circumstances.

Both authors carefully read all responses several times to familiarize themselves with the range of reasons respondents gave for their SRH ratings. Following principles of inductive qualitative content analysis, the initial category-development phase was treated as an exploratory step aimed at identifying recurrent types of reasons in the material. These types of reasons, which included a variety of physical, psychological, social and lifestyle-related aspects, were then condensed into a first draft of the coding scheme with 12 sub-categories. After a pilot coding of the first 70 responses jointly by both authors, the coding scheme was discussed and slightly revised to eliminate ambiguities in category assignment. For instance, in this stage, we separated “motivational” from “affective” psychological reasons and “healthcare system” from “society-related” reasons; moreover, we also added “alcohol and tobacco” as well as “check-ups” to the lifestyle domain. Table 1 documents the final coding scheme, including anchor examples for each category. Both authors then independently coded the full data set using the final coding scheme. Interrater reliability, assessed with Cohen’s kappa, was 0.87 [35], indicating a very high level of agreement [36, 37]. The remaining discrepancies were discussed and resolved by consensus. After defining and coding the categories, we additionally also coded the valence of each response, following Garbarski et al. [2], who distinguished between positively valenced and negatively valenced responses, ambivalent responses (containing both positive and negative tones), and neutral responses without a discernible affective tone.

**Table 1:** Coding scheme for categorization of open-ended responses

Biological factors	Psychological factors	Social factors	Lifestyle factors
**B1** Reference to (lack of) **acute illnesses or bodily symptoms**, such as back pain or headache. *Examples: “Nothing hurts*,* no pain.”; “I’m constantly catching colds.”*	**P1** Reference to positive or negative **affective states**, such as joy or sadness. *Examples: “I am almost always in a good mood.”; “I am happy*,* that’s the most important thing.”*	**S1** Reference to **interpersonal relations**, such as family relationships, friends and peers. *Example: “Support and harmony within the family nourish my well-being.”*	**L1** Reference to **nutritional behavior**, such as eating a vegetable-rich diet. *Examples: “I live vegan.”; “I don’t eat fast food or food containing additives.”*
**B2** Reference to **chronic conditions or illnesses**, such as asthma or osteoarthritis. *Examples: “I have chronic pain after an accident.”; “My chronic illnesses are getting worse every year.”*	**P2** Reference to **motivational states**, such as disinterest, excitement or having energy. *Examples: “I often feel tired and lacking in inner drive.”; “I am eager to try new things and have lots of interests.”*	**S2** Reference to **work- and income-related conditions**, such as pressure to perform or financial strain. *Examples: “I’m doing shift work for years.”; “I have a secure job – that matters.”*	**L2** Reference to **sporting behaviors and physical activities**, such as running, cycling or walking. *Examples: “I make sure to exercise regularly.”; “I cycle a lot*,* sometimes 75 km a day.”*
**B3** Reference to **physical abilities**,** functional limitations** or bodily constitution. *Examples: “I have a disability – my mobility is constrained.”; “My eyesight is very poor.”*	**P3** Reference to **cognitive abilities and limitations** or a specific state of mind. *Example: “I am mentally clear-headed and aware of my thoughts and surroundings.”*	**S3** Reference to **society-related conditions**, such as political developments and societal crisis. *Example: “I feel frustrated by the political situation.”*	**L3** Reference to **consumption of alcohol and tobacco** and other drugs. *Examples: “Had too much alcohol over the years.”; “I don’t smoke*,* never did.”*
**B4** Reference to received **medical treatments or medications**, such as hospitalization or “taking pills”. *Example: “Had three surgeries the last 6 months.”*	**P4** Reference to **mental health diagnoses or treatments**, such as depression or being in a psychotherapy. *Example: “I suffer from a major depression.”*	**S4** Reference to **healthcare and the healthcare system**, such as quality of services and received treatments. *Example: “No more medical specialists in my region.”*	**L4** Reference to preventive **medical check-ups and screenings** and their results. *Example: “I go for regular medical screenings – no signs of anything.”*

It needs to be acknowledged that qualitative coding always involves choices about the categories and the appropriate level of abstraction. A variety of alternative coding schemes have been applied in previous studies [32]. Moreover, coding would have been possible at different levels of abstraction, for example by collapsing the categories into broad biopsychosocial domains or by distinguishing more fine-grained disease-specific reasons. We chose the present intermediate level of abstraction because it preserved meaningful differences in respondents’ explanations while remaining sufficiently parsimonious and reliable for short open-text responses. In addition, about 12% of responses were coded as “other.” We assigned to this category those responses that were highly imprecise or idiosyncratic and showed no discernible connection to the coding scheme. Many responses in this category are open to interpretation: for instance, a response such as “*I have no complaints*” does not allow to infer whether it refers to mental or physical aspects of health. The same ambiguity applies to statements such as “*Nothing serious to report*”. Nevertheless, we incorporated selected responses from this category into our qualitative findings.

We analyzed the data as follows: We first provide a descriptive overview over the frequencies of each category, showing which reasons are most prevalent for justifying health ratings. Second, we are interested in comparing the reasons for SRH between different subgroups of individuals, particularly those with “good” and “poor” self-ratings. Therefore, we combined fair, poor and very poor SRH into one category (“poor”) and good and very good SRH into another (“good”). Given that only a small proportion rated their health as “poor” or “very poor”, these subgroups are too small to be reported separately. Third, we also compared differences according to socio-demographics, namely age, gender and SES. These comparisons are based on logistic regression analyses with the dummy-coded category as the outcome variable and SRH, gender, age, education and income as predictors. We performed a regression analysis for each category that was mentioned at least by 50 respondents. These differentiated analyses show whether individuals from different social groups justify health ratings with the same or with different criteria. Fourth, we analyzed whether individuals refer to one-dimensional reasons (e.g., only physical aspects) or multi-dimensional reasons (e.g., biopsychosocial aspects) when justifying their health ratings. Fifth, we exploited the study’s mixed methods design by illustrating the nuances in the open-ended question and between the subgroups by quoting some typical original responses.

## Results

### Justification for health-related self-ratings

**Fig. 1:**
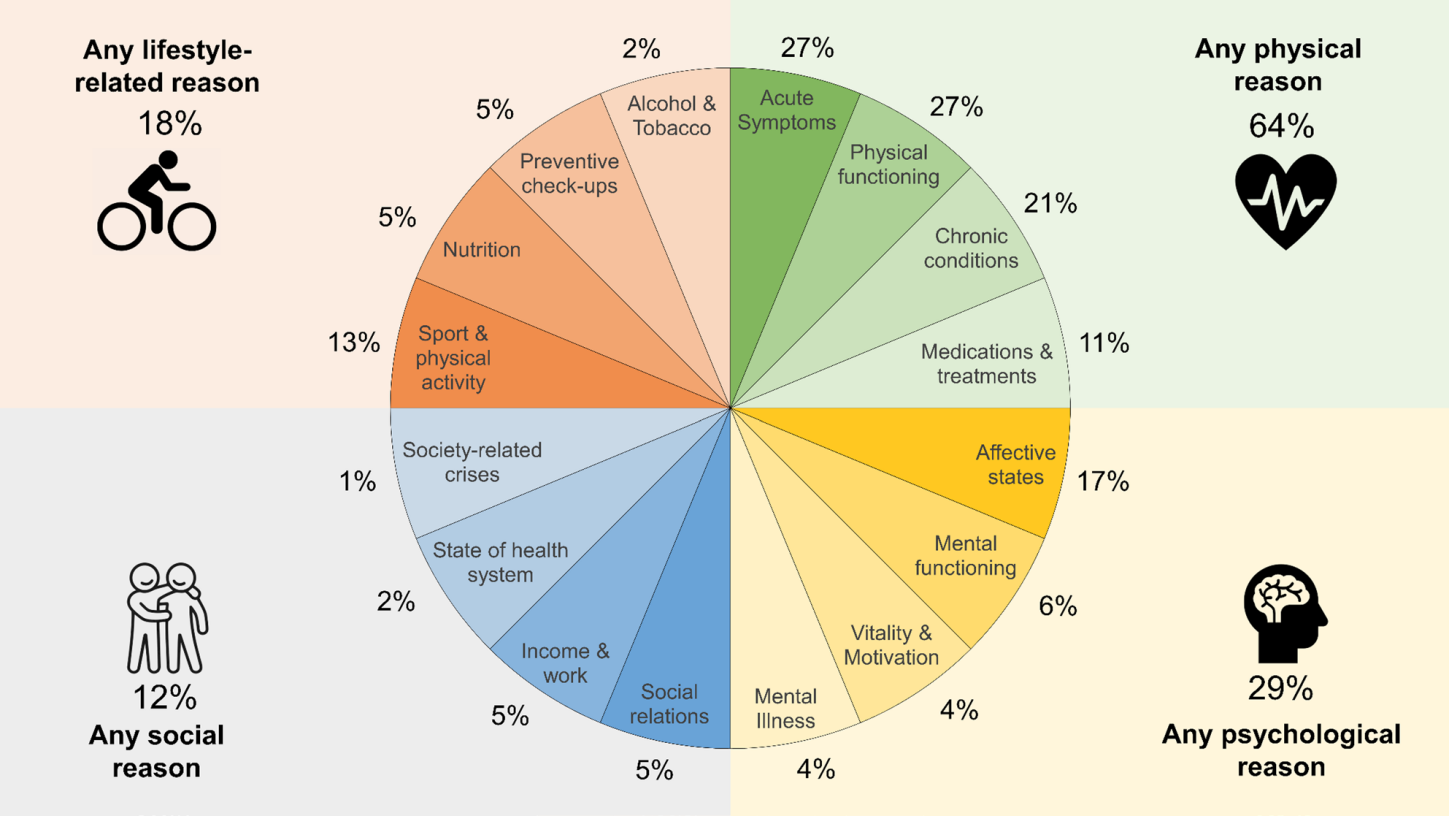
Frequency distribution of the reasons provided as justification for health-related self-ratings

Overall, the respondents mentioned 1.55 reasons, on average, to justify their health self-rating (*SD* = 1.08). Two thirds (64%) mentioned at least one physical aspect; 29% referred to at least one psychological aspect; 12% reported at least one social aspect, and 18% referred to at least one lifestyle-related aspect (Fig. 1). At a first glance, the stated reasons reveal a clear prioritization for the physical dimension of health and lend less relevance to psychological, social or lifestyle factors.

When single categories are analyzed, acute symptoms (27%) and physical functioning (27%) are most often cited by respondents to justify their SRH. Bodily symptoms, such as acute pain, as well as functional capacities, such as limitations in walking, climbing stairs, or sustaining work and household activities, are signals of compromised biological functioning. Both categories are followed by references to chronic conditions (20%), affective states (17%), sport and physical activity (13%), and medications and treatments (11%). Between 4% and 6% of the respondents mention mental functioning, social relations, work- and income-related reasons, preventive check-ups, their nutritional behaviors, vitality and motivation or mental illness. Only a small number of responses (1% to 2%) refer to the state of the health system, to alcohol, tobacco and other drugs or to societal issues and crises.

### Differences between “good” and “poor” health

Logistic regression analyses (Table 2) indicate that individuals with “poor” SRH refer significantly more often to chronic conditions to justify their rating; the odds ratio [OR] was 4.80. Typical statements of respondents with poor SRH illustrate the burden of a chronic illness, such as, “*Arthrosis in shoulders and knees makes pain-free movement impossible*”. In addition, respondents with poor SRH mentioned significantly less often physical functioning [OR = 0.34], sport and exercise [OR = 0.13], check-ups [OR = 0.12] and affective states [OR = 0.09].

**Table 2:** Logistic regression analyses for the 10 most frequently cited reasons for health-related self-ratings

	1	2	3	4	5	6	7	8	9	10
	Acute Symptoms	Physical functioning	Chronic conditions	Affective states	Sport & exercise	Treatments/ medication	Mental functioning	Preventive Check-ups	Social Relations	Work & Income
Self-rated health										
fair/poor/very poor (vs. very good/good)	1.18	0.34*	4.80**	0.09**	0.13**	1.11	0.83	0.12**	1.12	0.83
Gender										
female (vs. male)	0.98	0.83	1.00	1.61*	0.97	0.84	1.33	0.78	2.12*	2.14*
Age										
18–29 years (vs. ≥60)	2.45**	0.97	1.33	0.59	0.42*	0.09**	1.00	0.34*	2.37^+^	0.41
30–44 years	2.08**	0.67^+^	1.42	0.95	0.76	0.34**	1.53	0.12**	0.96	1.20
45–59 years	1.34	0.53**	0.98	0.75	0.46*	0.69	0.75	0.79	0.35^+^	0.97
SES										
Higher secondary (vs. medium/lower secondary degree)	1.00	1.15	1.25	0.65^+^	1.68*	0.99	1.41	1.35	0.70	0.85
> 4.000 EUR income (vs. ≤ 4.000 EUR)	1.05	1.51*	0.50**	1.69*	1.09	1.46	0.72	1.45	1.13	1.76
Nagelkerke R²	0.035	0.095	0.172	0.165	0.122	0.070	0.020	0.124	0.058	0.042

Table shows Odds Ratios

Significance: +*p*<.10; *p<.05; **p<.01. Significant effects (p<.05) are in bold

The open-ended responses show that health-related lifestyle factors, such as playing sports or taking regular medical checks, are almost without exception used as a positive argument to back a good health rating and almost never as a negative reason for poor health. This pattern is most evident in relation to exercise behavior, where respondents often mention the frequency of sporting activities (“*I am doing fitness in a gym three times a week*”) or refer to sporting routines (“*I go for a 10 km walk each day*”). Likewise respondents refer to nutrition, justifying their good state of health with “*healthy*,* balanced meals*” or “*cooking fresh food every day*,* partly from the garden*”. Affective states are also more often used as a justification for a good overall health rating. Typical statements included, for instance, “*I feel very well at the moment*” or “*I feel good*,* I’m happy and I can do whatever I want*”.

Regarding valence, we found that justifications for good vs. poor SRH scores differ strongly in their positive and negative tone: Among respondents with good SRH, 81% of responses were positive, 5% negative, 8% ambivalent and 6% neutral. Among respondents with poor SRH, in contrast, 3% of responses were positive, 92% negative, 1% ambivalent and 4% neutral. This large difference between groups was statistically significant, χ²(3, 854) = 656.3, *p*<.001, Cramer’s V = 0.88.

### Differences according to gender

Women compared to men justify health significantly more often with respect to affective states [OR = 1.61], interpersonal relations [OR = 2.12], or work and income [OR = 2.14]. Typical examples include to “*feel very well mentally*” or “*I know what I need to do to maintain a positive feeling*” or “*well-being is the most important thing; pay attention to your body’s signals*”. Typical responses that entail a gender-specificity refer to childcare and family obligations, such as “*children are constantly ill*,* which worries me*” or “*overworked from job and family*,* I don’t have time for self-care*”. In addition, care work is sporadically used as a reason for less than good health ratings, for instance, one woman stated: “*I have been caring for my husband*,* who requires long-term care*,* all by myself*,* day and night*,* for three years!*” In sum, women’s health ratings appear to be more relational and affectively grounded.

### Differences between age groups

Younger respondents compared to older respondents justify health significantly more often with respect to acute states; the OR for 18- to 29-year-olds is 2.45 and for 30- to 44-year-olds 2.08. Typical statements from younger respondents either refer to acute symptoms (“*I have a feeling that I’m coming down with a cold*”) or to their absence (“*no acute illnesses*”, “*no acute symptoms*,* I am rarely ill*”). In addition, younger cohorts, for instance 18- to 29-year-olds, significantly less often state received medical treatments, diagnoses or medications [OR = 0.09] as well as preventive check-ups [OR = 0.34] as reasons for their health ratings. Middle-aged respondents (45- to 59-years) mention physical functioning [OR = 0.53] as well as sport and exercise [OR = 0.46] less often than older respondents (≥ 60 years). It seems though that bodily functions and the ability to play sport and exercise are taken-for-granted by young and middle-aged cohorts, whereas respondents over 60 years of age often emphasize these as a proof for being in a good state of health. Sometimes older respondents refer to their sporting abilities in combination with their age to justify their health, such as, “*I am still able to play soccer*”, “*I can still do all my favorite sporting activities*” or “*I play table tennis with friends once a week and can still compete with the younger ones*”.

Compared to older individuals (≥ 60 years) the youngest age group refers more often to social relations (OR = 2.37), whereas middle-aged respondents refer less often to relationships (OR = 0.35). The link between social relationships and health is drawn, for instance, by referring to “*a well-balanced social life*” or “*mutual support and harmonious social interaction*”. Most often, the family is the reference of such reasons.

It is noteworthy how many individuals explicitly refer to age in their open-ended responses. Age is often used as a key anchor point for perceiving, emphasizing and evaluating one’s condition, as in statements such as “*Despite my age*,* I can still do everything*” or “*Considering my age*,* I have few complaints*”. Furthermore, older respondents frequently refer to age alone as the reason for a poor self-assessment, simply stating *“my age”* or *“old age”*. In contrast, younger respondents sometimes refer to their young age in their responses to justify their positive self-assessment: *“I am young*,* so I am not limited by my age”.* This suggests that some people consider age itself as a sufficient reason for a health rating. Similarly, health assessments are also related to one’s own biography, for example: “*I’m 70 now – nothing works the way it did 10 years ago*” or “*Since I’ve been in a wheelchair*,* I can no longer enjoy life as much as I once did*”. In this sense, SRH appears to reflect not only present health but also how individuals position themselves within the ageing process and their life course.

### Differences according to SES

Higher education is associated with more referrals to sport and exercise [OR = 1.68]. Higher income is associated with fewer referrals to chronic conditions [OR = 0.50], but with more referrals to affective states [OR = 1.69]. For instance, individuals with a higher SES described their life as “*a good balance between work performance and relaxation*” or stated “*I feel good*,* I like my work*,* it’s fun*”. The open-ended questions further illustrate that many individuals with a higher SES associate health with autonomy, independence and the capacity to work productively. Typical statements are “*I’m in charge of my life*”, “*I can participate in life without any restrictions*” or *“I can still do my demanding job despite the first signs of aging”*. Compared to other social groups, health is more often seen as an achievement or a merit. One respondent, for instance, explicitly justifies a very good health-rating with “*personal responsibility and self-discipline*”, while other respondents claim “*I take good care of myself*” or *“I’m fit and working to make sure I stay that way for a long time”.* In this sense, health-related rationales are also a marker of self-control and competence.

### Personal health in relation to the health of others

Interestingly, the “social relations” category included not only statements about the quantity or quality of social relationships and social support, but also, in some cases, references to significant others in the family or among friends as direct comparison points in the assessment of one’s own health. For instance, one typical statement reads: “*Overall*,* I’m happy with my health compared to that of my friends and acquaintances*.” In addition to using others as a reference point, respondents sometimes also incorporated the health of close relatives into their assessment of their own health. For example, reasons given for a positive self-assessment included statements such as *“healthy family”* or *“my family and I are doing well”*, whereas reasons for a negative self-assessment included statements such as “*my wife is not doing so well.*” This suggests that, for some people, subjective health encompasses more than their own individual health and is genuinely relational in nature.

### One- vs. multidimensional reasons for SRH

A large majority of 66% provided a one-dimensional reason for their health rating. However, a one-dimensional justification may still include more than one reason. For instance, respondents frequently referred to several lifestyle aspects to justify their health: Nutrition and sport were frequently mentioned together (*r* = .53), but also nutrition and the consumption of alcohol and tobacco (*r* = .27) as well as sport and the consumption of alcohol and tobacco (*r* = .23).

A minority of 34% provided a multi-dimensional justification that is more representative for a biopsychosocial reasoning. Multi-dimensional justifications are less common among individuals with poor SRH compared to those with good health ratings (OR = 0.32). Unlike poor health, good health is more often understood not just in terms of physical illness, but as a biopsychosocial state of well-being. For instance, one respondent wrote: “*I rate my health as very good because I feel physically and mentally balanced. I eat a healthy diet and exercise regularly. My family also plays a big role. Doing activities together contribute greatly to my well-being*”. Women are also more likely than men to refer to multidimensional reasons to justify their health (OR = 1.52).

Some bivariate correlations between categories were significant (*p* < .05), however most of them are negative. For instance, individuals who mention chronic conditions are less likely to mention affective states (*r* = − .18) and social relations (*r* = − .12). Individuals who refer to acute symptoms are less likely to mention nutritional behaviors (*r* = − .13) or preventive check-ups (*r* = − .12). Few correlations point to positive cross-dimensional associations, i.e. that categories from different domains are likely to be mentioned in the same response: Individuals who mention physical functioning are more likely to mention mental functioning as well (*r* = .10). A typical response that combines both is “*No physical limitations*,* and mentally I seem to be fine as well*”. Individuals who mention (lack of) vitality and motivation are more likely to mention work-related reasons (*r* = .12) or societal issues and crisis (*r* = .12). For instance, respondents refer to “*feeling constantly exhausted at work*” or to “*the psychological strain because of these incompetent politicians makes me sick*”. In view of only few and small correlations, it seems that single reasons reflect relatively discrete dimensions of experience with limited overlap across domains.

## Discussion

The present research set out to uncover the reasons that respondents use when they justify a single-item assessment of their health and to examine how these subjective justifications differ across key sociodemographic strata. The use of an open‑ended prompt embedded in a large‑scale, representative survey allowed us to explore inasmuch self‑rated health (SRH) is not a unitary construct but a synthesis of reasoning related to biological, psychological and social information [1, 2]. The findings shed light on the relative weight of each domain and help to understand inasmuch individuals rely on more than one domain when assigning their health. Moreover, findings show how the weight of each domain varies by health status, and on its distribution across age, gender and socioeconomic status (SES). They also raise critical methodological and practical questions for future research and health‑policy practice.

We would like to carve out several key findings: In line with previous research [19–22, 28], physical symptoms dominate the justification universe. Reasons for SRH are dominated by referrals to acute and chronic biological conditions as well as physical functioning. The strong influence of biological cues on SRH suggests that lay people do not frequently measure their health against a biopsychosocial model, but are self‑classifying primarily by bodily states rather than psychosocial context. In addition, justifications for poor SRH were mostly negatively valenced, whereas reasons for good SRH had predominantly a positive tone. The analysis of valence buttressed previous findings [2]. Going beyond previous research, we found that the combination of arguments from different domains of health was depending on the SRH rating. Individuals who rated their health less-than-good were far more likely to provide one-dimensional responses, usually solely referring to the biological dimension of health. In case of very good health, in contrast, people tend to weave together biological, psychological, social and lifestyle cues and more often justify their ratings with multi-dimensional responses. In other words, poor health is largely justified and understood as biological, whereas good health was more often framed in biopsychosocial terms.

Previous research has already pointed out that the meanings behind SRH vary across socio-demographic groups [2, 28–33]. The present study also found age-, gender-, and SES-specific patterns, but not everywhere. Women are more inclined to refer to emotions, interpersonal relationships and work‑related pressures than men. Similar findings were shown for mothers shortly after childbirth [34], but not women in general. Young adults focus more on acute symptoms and social life; they mention chronic conditions and medical care far less often than older adults. Higher education is linked to greater mention of sport and exercise, while higher income is associated with fewer references to chronic illness and a greater focus on mood and personal satisfaction. Respondents from the high SES groups focus on behavioral choice and autonomy and see health as a merit resulting from self-discipline and self-care.

Social relations, which were somewhat neglected in previous research on SRH, figure in in two ways: They function as a resource for support, well-being and care, but also as a reference to evaluate personal conditions against a social benchmark. In this respect, our findings are consistent with previous findings that SRH is formed through comparison with a variety of personally relevant social groups and benchmarks [30]. Other reference points for justifying health evaluations are “objective” medical criteria (e.g., *high blood pressure*), biographical criteria (e.g., *compared to 10 years ago*…) and age-related criteria (e.g., *for someone in his 70ies*…).

With regard to the biopsychosocial model the shift from single‑dimension to multi‑dimensional justification with improved health matches a dynamic comparison process, where the salience of each domain depends on the relative deviation from one’s perceived personal norm. Previous studies [5, 30] imply that SRH is not based on a fixed checklist, but that individuals compare their current situation to their own personal norm or expectation. It seems highly plausible though, that a domain becomes salient when it deviates noticeably from what the person considers ‘normal’ for themselves. For instance, if someone has a strong physical problem, the physical domain dominates the rating. If there is no major problem in one domain, people may draw on several domains at once to describe why they feel healthy overall. This interpretation is consistent with broader theories of judgment and appraisal, which assume that evaluations are made relative to personal reference points, adaptation levels and multiple comparison standards rather than fixed absolute criteria [38, 39].

Age‑specific patterns are likely to reflect developmental and role changes. The younger group’s focus on acute symptoms and social relations indicates a health evaluation heavily influenced by current experiences and social network quality. In contrast, older respondents tie health to chronic disease burden and functional capacity, consonant with the life‑course perspectives on the cumulative effects of health-relevant exposures across the lifespan [40]. From an accumulation perspective, these age differences suggest that SRH in later life may be increasingly shaped by the long-term layering of illness and functional decline. In addition, gender differences, especially women’s heightened attention to affective states, social relations and caregiving responsibilities, may echo gender role-based theories of health [41].

Higher-income groups refer less often to chronic disease but more often to emotional well-being, reflecting a protective effects of material and social resources. Conversely, lower-income respondents’ stronger emphasis on chronic conditions likely mirrors the unequal distribution of disease burden and long-term health risks [42]. With higher SES, levels of health knowledge, health literacy, prevention, and use of preventive care tend to increase. At the same time, expectations towards the health-care system appear to be more demanding, while one’s own role is understood as active, informed, and competent in maintaining health [43]. These patterns illustrate that SRH is not merely a measure of health status, but also a socially situated statement shaped by access to resources, knowledge, and agency.

### Strengths and limitations

One strength of our study lies in its mixed-methods design, which allowed us to combine the openness of qualitative inquiry with the generalizability of quantitative research. The open-ended question without predefined answers enabled participants to articulate their evaluations in their own terms and according to their own systems of relevance. This is a particular advantage of open-ended formats: rather than forcing respondents into researcher-defined categories, they make it possible to capture unanticipated meanings and the language through which people themselves interpret health. As a result, they are especially well suited to uncovering the underlying interpretive schemes that structure subjective health ratings. At the same time, because these qualitative insights were generated from representative survey data, we were also able to examine how such patterns vary systematically across social groups. The mixed-methods approach therefore not only enriches our understanding of what respondents mean when they rate their health, but also allows these meanings to be situated within broader population-level patterns.

However, several limitations should be noted. First, our design entails an inherent trade-off between depth and representativeness. Small-scale qualitative interviews can probe the reasoning behind health assessments in considerable depth, but typically lack representativeness. Large-scale representative surveys, by contrast, achieve population coverage at the cost of depth, as respondents often provide only short and concise answers to open-ended questions. Our study clearly falls on the representativeness side of this trade-off: the brevity of many responses likely constrained the extent to which respondents elaborated on the reasons underlying their health assessments. This may have encouraged participants to report only the most salient or immediately accessible cue, thereby under-representing the multidimensionality that may also inform subjective health ratings. In other words, some respondents may well have drawn on several domains when evaluating their health, but articulated only one in the survey context.

Second, the possibility of response bias must be considered. The willingness or ability to provide a written explanation may be socially patterned: respondents with higher levels of education, greater health literacy, or stronger verbal and written skills may have been more likely to offer fuller or more differentiated answers. Conversely, individuals with more limited literacy or expressive capacity may be under-represented in the more detailed responses, which could systematically shape the distribution and content of the justifications we observed. This constraint may also apply to immigrant groups with limited German language proficiency.

Third, our study did not collect objective health data, such as clinical measurements or medical records. Subjective ratings can therefore not be validated against objective indicators of health status. This limitation is particularly relevant for the interpretation of subgroup differences. While our analysis identifies systematic variation in the reasons respondents give for their SRH ratings across social groups, the present design cannot fully disentangle whether these differences reflect variation in the cognitive and interpretive processes underlying SRH or differences in actual health status between groups. Both mechanisms are plausible and not mutually exclusive: groups may differ in the health conditions they are exposed to *and* in the interpretive frames through which they evaluate their health. Future research combining open-ended SRH probes with objective health measures would be well placed to separate these components more clearly. At the same time, our findings retain their value as evidence on the reasons respondents themselves invoke when explaining their health, which is informative regardless of how these reasons map onto objective health states.

Taken together, these limitations suggest that the reported patterns should be interpreted as reflecting the reasons respondents chose and were able to articulate, rather than as a complete account of all considerations entering their SRH judgments.

## Conclusion

This study demonstrates that the widely used single‑item SRH measure is not a monolithic judgement of health. Rather, it is a nuanced, domain‑weighted judgment that varies systematically with health status, age, gender, and socioeconomic position. Although bodily complaints dominate many respondents’ justifications, ‘very good’ health is more often described in broader, biopsychosocial terms, indicating that the meaning of SRH shifts with circumstances. SRH should therefore be understood neither as a simple proxy for biological health alone nor as a consistently integrated assessment of all relevant dimensions of health. Instead, our findings support a more context-sensitive interpretation: respondents draw on different domains of health depending on which aspects are most salient in light of their current condition, well-being, social roles, and broader life situation. Recognizing this variability is essential for the interpretation of SRH in research and public health, because the same survey item may capture different underlying evaluative processes across population groups.

## Supplementary Information

Supplementary Material 1.

## Data Availability

The dataset supporting the study findings is available from the corresponding author upon reasonable request.
