# Supplementary material for: Reasons behind individuals’ self-ratings of health: an analysis of responses to an open-ended survey question
Source: BMC Public Health. 2026 May 23;26:1682. doi: 10.1186/s12889-026-27905-0 (PMC13198053; doi:10.1186/s12889-026-27905-0)
Supplement: Supplementary file 1 — Supplementary Material 1. [file 12889_2026_27905_MOESM1_ESM.pdf]

Supplement.

Reasons behind individuals' self-ratings of health: An analysis of responses to an open-ended survey question

Questionnaire Wording

1. How is your health in general?

Very poor	poor	fair	good	Very good
O	O	O	O	O

2. You have just rated your health as [very poor | poor | fair | good | very good].

   What are the most important reasons for this self-assessment?

